# Supplementary material for: Temporal Structure of Item Presentation Modulates Brain Oscillations in Verbal Working Memory
Source: Eur J Neurosci. 2025 Dec 29;63(1):e70371. doi: 10.1111/ejn.70371 (PMC12746538; doi:10.1111/ejn.70371)
Supplement: Supplementary file 1 — Figure S1: Results of cluster‐based permutation tests across all electrodes for the alpha (9–13 Hz), theta (4–7 Hz), and beta (18–24 Hz) frequency bands during (1) encoding, (2) retention 1 (0.6–3 s), and (3) retention 2 (3–6 s). The color scale represents t‐values, with positive values indicating stronger activation in the presentation mode listed on the right compared with the condition listed at the top (e.g., Fast vs. Simultaneous for alpha during encoding). Red dots indicate electrodes showing significant differences between the compared conditions. Only significant effects are displayed. [file EJN-63-0-s001.pdf]

Supplementary Results

Cluster-based permutation tests were run separately for each frequency band and task period, comparing presentation modes pairwise. The analysis was conducted in Fieldtrip toolbox (Oostenveld et al., 2011) with 5,000 permutations, a cluster alpha of 0.05, and a minimum cluster size of 2 neighboring channels.

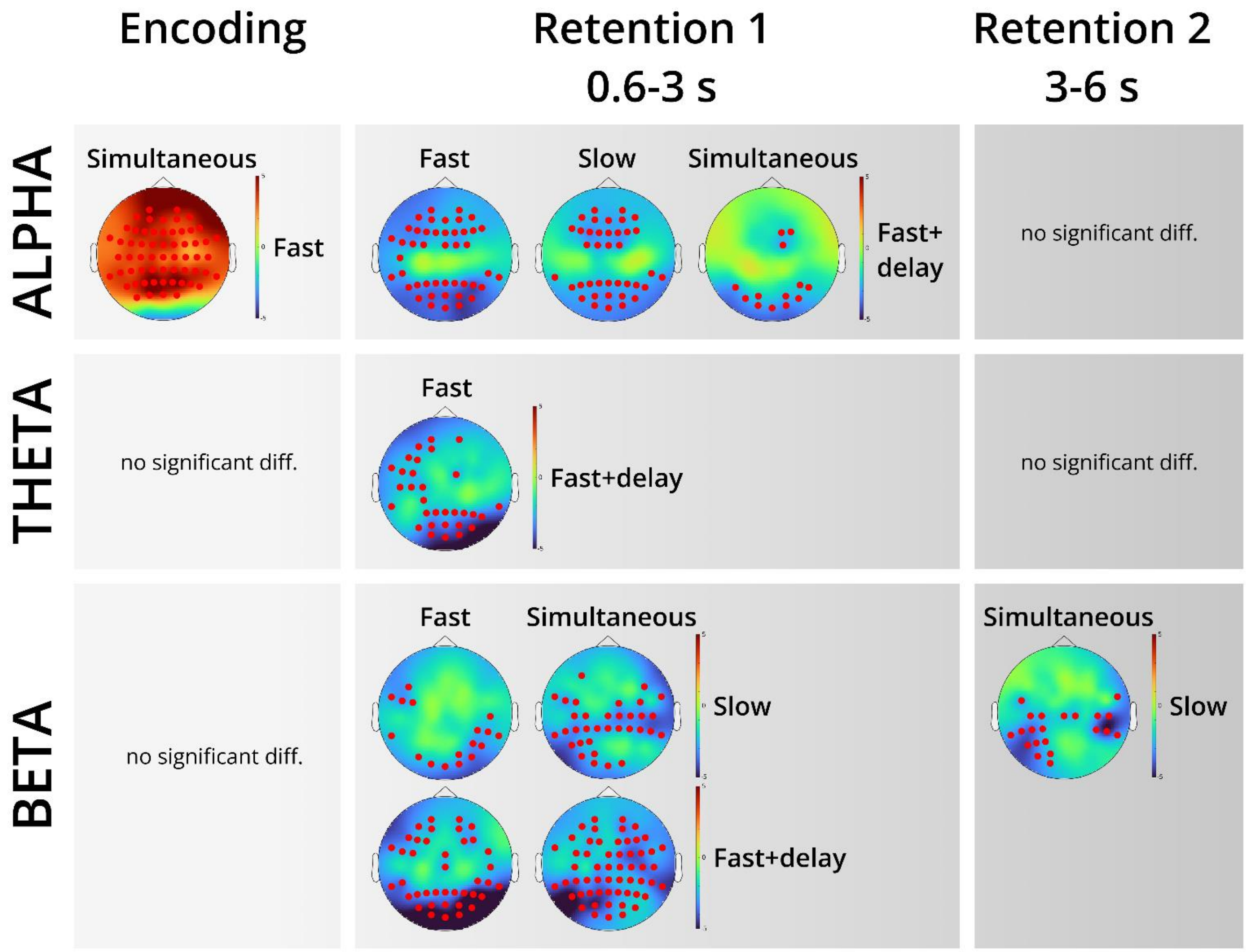

Figure S1. Results of cluster-based permutation tests across all electrodes for the alpha (9-13 Hz), theta (4-7 Hz), and beta (18-24 Hz) frequency bands during (1) Encoding, (2) Retention 1 (0.6-3 s), and (3) Retention 2 (3-6 s). The color scale represents t-values, with positive values indicating stronger activation in the presentation mode listed on the right compared with the condition listed at the top (e.g., Fast vs. Simultaneous for alpha during encoding). Red dots indicate electrodes showing significant differences between the compared conditions. Only significant effects are displayed.
